# Safety of immune checkpoint inhibitors in patients aged over 80 years: a retrospective cohort study

**DOI:** 10.1007/s00262-024-03707-4

**Published:** 2024-05-11

**Authors:** Tatsuki Ikoma, Toshihiko Matsumoto, Shogen Boku, Yusuke Motoki, Hidefumi Kinoshita, Hisashi Kosaka, Masaki Kaibori, Kentaro Inoue, Mitsugu Sekimoto, Takuo Fujisawa, Hiroshi Iwai, Makoto Naganuma, Hideaki Tanizaki, Yoji Hisamatsu, Hidetaka Okada, Takayasu Kurata

**Affiliations:** 1 Cancer Treatment Center, Kansai Medical University Hospital, 2-3-1, Shinmachi, Hirakata, Osaka-Prefecture 573-1191 Japan; 2 Department of Thoracic Oncology, Kansai Medical University, 2-3-1, Shinmachi, Hirakata, Osaka-Prefecture 573-1191 Japan; 3 Department of Urology and Andrology, Kansai Medical University, 2-3-1, Shinmachi, Hirakata, Osaka-Prefecture 573-1191 Japan; 4 Department of Surgery, Kansai Medical University, 2-3-1, Shinmachi, Hirakata, Osaka-Prefecture 573-1191 Japan; 5 Department of Otorhinolaryngology, Head and Neck Surgery, Kansai Medical University, 2-3-1, Shinmachi, Hirakata, Osaka-Prefecture 573-1191 Japan; 6 Third Department of Internal Medicine, Kansai Medical University, 2-3-1, Shinmachi, Hirakata, Osaka-Prefecture 573-1191 Japan; 7 Department of Dermatology, Kansai Medical University, 2-3-1, Shinmachi, Hirakata, Osaka-Prefecture 573-1191 Japan; 8 Department of Obstetrics and Gynecology, Kansai Medical University, 2-3-1, Shinmachi, Hirakata, Osaka-Prefecture 573-1191 Japan

**Keywords:** Immune checkpoint inhibitor, Immune-related adverse events, Older patients, Eosinophil, Albumin

## Abstract

**Background:**

Immuno-oncology (IO) drugs are essential for treating various cancer types; however, safety concerns persist in older patients. Although the incidence of immune-related adverse events (irAEs) is similar among age groups, higher rates of hospitalization or discontinuation of IO therapy have been reported in older patients. Limited research exists on IO drug safety and risk factors in older adults. Our investigation aimed to assess the incidence of irAEs and identify the potential risk factors associated with their development.

**Methods:**

This retrospective analysis reviewed the clinical data extracted from the medical records of patients aged > 80 years who underwent IO treatment at our institution. Univariate and multivariate analyses were performed to assess the incidence of irAEs.

**Results:**

Our study included 181 patients (median age: 82 years, range: 80–94), mostly men (73%), with a performance status of 0–1 in 87% of the cases; 64% received IO monotherapy. irAEs occurred in 35% of patients, contributing to IO therapy discontinuation in 19%. Our analysis highlighted increased body mass index, eosinophil counts, and albumin levels in patients with irAEs. Eosinophil count emerged as a significant risk factor for any grade irAEs, particularly Grade 3 or higher, with a cutoff of 118 (/μL). The group with eosinophil counts > 118 had a higher frequency of irAEs, and Grade 3 or higher events than the group with counts ≤ 118.

**Conclusion:**

IO therapy is a safe treatment option for patients > 80 years old. Furthermore, patients with elevated eosinophil counts at treatment initiation should be cautiously managed.

## Introduction

Immuno-oncology (IO) drugs have been employed as standalone treatments and established as the standard treatment for advanced cancer [1]. There are two primary types of immune checkpoint blockade: anti-PD-1/PD-L1 inhibitors (targeting the programmed cell death protein 1 and its ligand, PD-L1) and anti-CTLA-4 inhibitors (targeting the cytotoxic T-lymphocyte-associated protein 4), as well as various other immune checkpoint blockades. Representative drugs for anti-PD-1/PD-L1 inhibitors include nivolumab, pembrolizumab, atezolizumab, and durvalumab, while ipilimumab is a well-known anti-CTLA-4 inhibitor (1). Moreover, their efficacy has been proved in combination therapy with cytotoxic anticancer drugs or targeted molecular agents, becoming an integral part of the treatment for various cancer types [2–4]. However, IO drugs exhibit a distinct adverse event profile compared to traditional cytotoxic anticancer drugs [5]. Therefore, managing immune-related adverse events (irAEs) is a crucial aspect of IO therapy [6–8].

An increasingly prevalent issue in modern cancer treatment is the high proportion of older patients with cancer. Although extensive randomized trials assessing the effectiveness and safety of immune checkpoint inhibitors in older adults and patients with functional impairment (performance status, PS) are lacking, a few reports have offered insights into this topic [9]. For instance, in a previous trial for non-small cell lung cancer (NSCLC), nivolumab did not increase the incidence of irAEs in patients aged ≥ 70 years or those with PS ≥ 2 or higher [9]. Similarly, a pooled analysis of several studies on pembrolizumab in NSCLC revealed that pembrolizumab was equally effective in older and younger patients, with better safety outcomes in older adults than in other chemotherapy recipients [10]. Furthermore, some studies have reported that the frequency of irAEs did not increase in patients who are physically vulnerable [11].

Chemotherapies, including IO drugs, are commonly administered to older patients in clinical practice; hence, identifying the risk factors for irAEs is crucial for determining populations that could genuinely benefit from effective and safe treatment. Hypoalbuminemia and type I hypersensitivity reactions have been reported as potential risk factors [12]. Additionally, no difference was observed in the incidence of irAEs between groups with and without frailty, as categorized by the Geriatric-8 tool; however, the report showed that patients who are physically vulnerable tended to have more hospitalizations and discontinuation of therapy due to AEs [13]. Furthermore, a multicenter cohort study evaluating IO monotherapy in patients aged ≥ 80 years found that the incidence of Grade 3 or higher irAEs according to the Common Terminology Criteria for Adverse Events version 5.0 (CTCAE) was 12.2% [14]. Although the incidence of irAEs does not increase with age, the discontinuation rate due to irAEs increases significantly [14]. Furthermore, there are reports indicating that the efficacy of treatment in elderly patients was comparable to that in non-elderly patients, with the incidence of irAEs being either similar to or lower than in younger patients [15, 16].

However, there is a dearth of studies investigating the incidence of irAEs and associated risk factors in older patients with solid tumors undergoing IO monotherapy or combination therapy. Therefore, this study aimed to assess the incidence of immune-related adverse events (irAEs) and identify potential risk factors associated with their development in older patients, potentially contributing to our understanding of irAEs in this population.

## Methods

### Study design and patient characteristics

The clinical data of consecutive patients with solid tumors who underwent IO therapy were retrospectively collected from our hospital. Eligible patients were aged ≥ 80 years and had received at least one cycle of a regimen with IO drugs between November 2014 and July 2022. The patient data were assessed from the date of registration to October 2023. The patients were administered anti-PD-1, anti-PD-L1, and anti-CTLA-4 antibodies, such as nivolumab, pembrolizumab, atezolizumab, durvalumab, avelumab, and ipilimumab until the end of the scheduled course, progression of tumor, or development of intolerance. Whether concomitant therapy included cytotoxic anticancer or molecular-targeted drugs was not assessed. Exclusion criteria include lack of consent, age of less than 80 years, and no patients with a solid tumor. The incidence and details of irAEs were assessed using data from the electronic medical records and laboratory test results. The severity of toxicity was categorized according to the guidelines for CTCAE 5.0. Additionally, data on the frequency of treatment-related hospitalizations and treatment discontinuation due to toxicity were collected. This study was conducted in accordance with the Helsinki Declaration of 1964 and its later versions and with the ethical guidelines for clinical studies. This study was approved by the institutional review board of our hospital (approval no. 2022218).

### Definition of nutritional factors

We investigated three factors: body mass index (BMI), neutrophil/lymphocyte ratio (NLR), and C-reactive protein (CRP)/albumin (Alb) ratio (CAR) [17]. These factors were obtained from the results of the physical examination and blood test on the date of the first IO therapy initiation. BMI was calculated using the following formula: BMI = (weight in kg)/(height in meters). NLR was defined as the absolute neutrophil count divided by the absolute lymphocyte count, and CAR was measured by dividing the serum CRP value by the serum Alb value.

### Statistical analysis

Fisher’s exact and Mann–Whitney U tests were used to compare patient characteristics. Univariate and multivariate logistic regression analyses were performed to assess the incidence of irAEs, and the odds ratios and 95% confidence intervals (CIs) were calculated. A receiver operating characteristic (ROC) curve was used to determine the correlation between the onset of irAEs and eosinophil counts, and the cutoff values were determined. The predictive performance was evaluated through the area under the ROC curve (AUC). Statistical analyses were performed using SPSS software (version 28.0; IBM Corp., Armonk, NY, USA), and statistical significance was set at *p* < 0.05.

## Results

### Patient characteristics

Between November 2014 and July 2022, 181 consecutive patients who were 80 years or older were treated with regimens including IOs, with a median observation period of 9.8 months (range: 0.3–72.8 months). The patients were divided into two groups depending on the occurrence of irAEs: no-irAE and yes-irAE, with 117 (65%) and 64 (35%) patients, respectively (Table 1). The yes-irAEs group had significantly fewer cases with Eastern Cooperative Oncology Group PS 2 or higher (*p* = 0.03). The most common primary lesions were in the thoracic region (39% and 34% in the no-irAE and yes-irAE groups, respectively). Most patients underwent IO monotherapy (62% and 67%, respectively), and combined immunotherapy was administered to 4% and 5% of the patients, respectively. No significant disparities were observed between the two groups, although comorbidity data were gathered exclusively for older participants.

**Table 1:** Patient characteristics of groups with irAEs (yes-irAEs) and without irAEs (no-irAEs)

Characteristics	*N* = 181 (%)			*p*-value
	All	No-irAEs *N* = 117	Yes-irAEs *N* = 64	
Age, years	82 [80–94]	82 [80–94]	82 [80–90]	–
Male, Sex	132 (73)	81 (69)	51 (80)	0.16
ECOG PS				
0	58 (32)	30 (25)	28 (44)	**0.03**
1	100 (55)	67 (57)	33 (52)	
2 or higher	23 (13)	20 (18)	3 (4)	
Primary lesion				
Head and neck	17 (9)	8 (7)	9 (14)	0.16
GI tract	22 (12)	11 (9)	11 (17)	
Liver	30 (17)	19 (16)	11 (17)	
Lung and pleura	68 (38)	46 (39)	22 (34)	
Genitourinary	34 (18)	27 (23)	7 (12)	
Skin	6 (3)	4 (4)	2 (3)	
The other	4 (2)	2 (2)	2 (3)	
Treatment				
IO monotherapy	116 (66)	72 (62)	43 (67)	0.52
IO + chemotherapy	24 (13)	19 (16)	5 (8)	0.17
IO + molecular target	34 (18)	21 (18)	13 (20)	0.69
IO + IO	8 (5)	5 (4)	3 (5)	1.00
Comorbidity				
HT	107 (59)	72 (62)	35 (55)	0.43
DM	38 (21)	24 (21)	14 (22)	0.85
CKD	34 (18)	24 (21)	10 (16)	0.55
CHF	29 (16)	17 (14)	12 (19)	0.53

ECOG PS, Eastern Cooperative Oncology Group performance status; IO, immune checkpoint inhibitor; GI, gastrointestinal; HT, hypertension; DM, diabetes mellitus; CKD, chronic kidney disease; CHF, chronic heart failure

Items with *p*-values in bold were less than 0.05

### Details of the onset of irAEs

The incidence of all grades of irAEs was 35%, with a 13% incidence of Grade 3 or higher events (Table 2). The most frequently occurring irAE was dermatitis (10%), and the most common Grade 3 or higher irAE was pneumonitis (3%). The toxicity-related discontinuation rate due to irAEs was 19%, and the treatment-related hospitalization rate was 5%. The irAEs that resulted in toxicity-related discontinuation were primarily pneumonitis (30%), followed by hepatitis (22%), colitis (9%), endocrine disorders (9%), nephritis (9% each), and dermatitis (4%). There were two cases of G5 toxicity and pneumonitis. Endocrine disorders included hypothyroidism in seven, type 1 diabetes in one, and hypoadrenocorticism in one. The median onset time for any irAEs was 53 days (range, 3–911 days). The median onset time was 86 days (range, 6–911 days) for dermatitis, 41 days (range, 7–275 days) for hepatitis, 85 days (range, 7–520 days) for pneumonitis, 74 days (range, 3–777 days) for colitis, 84 days (range, 21–361 days) for endocrine disorders, and 63 days (range, 55–63 days) for nephritis.

**Table 2:** Frequency of irAEs and toxicity details

Variable	*N* = 181 (%)	
	Any Grade	G3 +
irAE	64 (35)	23 (13)
irAE leading to discontinuation	23 (19)	
Specific irAE		
Dermatitis	19 (10)	0
Hepatitis	15 (8)	4 (2)
Pneumonitis	12 (7)	6 (3)
Colitis	9 (5)	4 (2)
Endocrine disorder	9 (5)	2 (1)
Nephritis	3 (2)	1 (1)
Other	13 (7)	4 (2)

G3 + ; Grade 3 or higher

### Examination of findings and risk factors associated with the incidence of irAEs

**Fig. 1:**
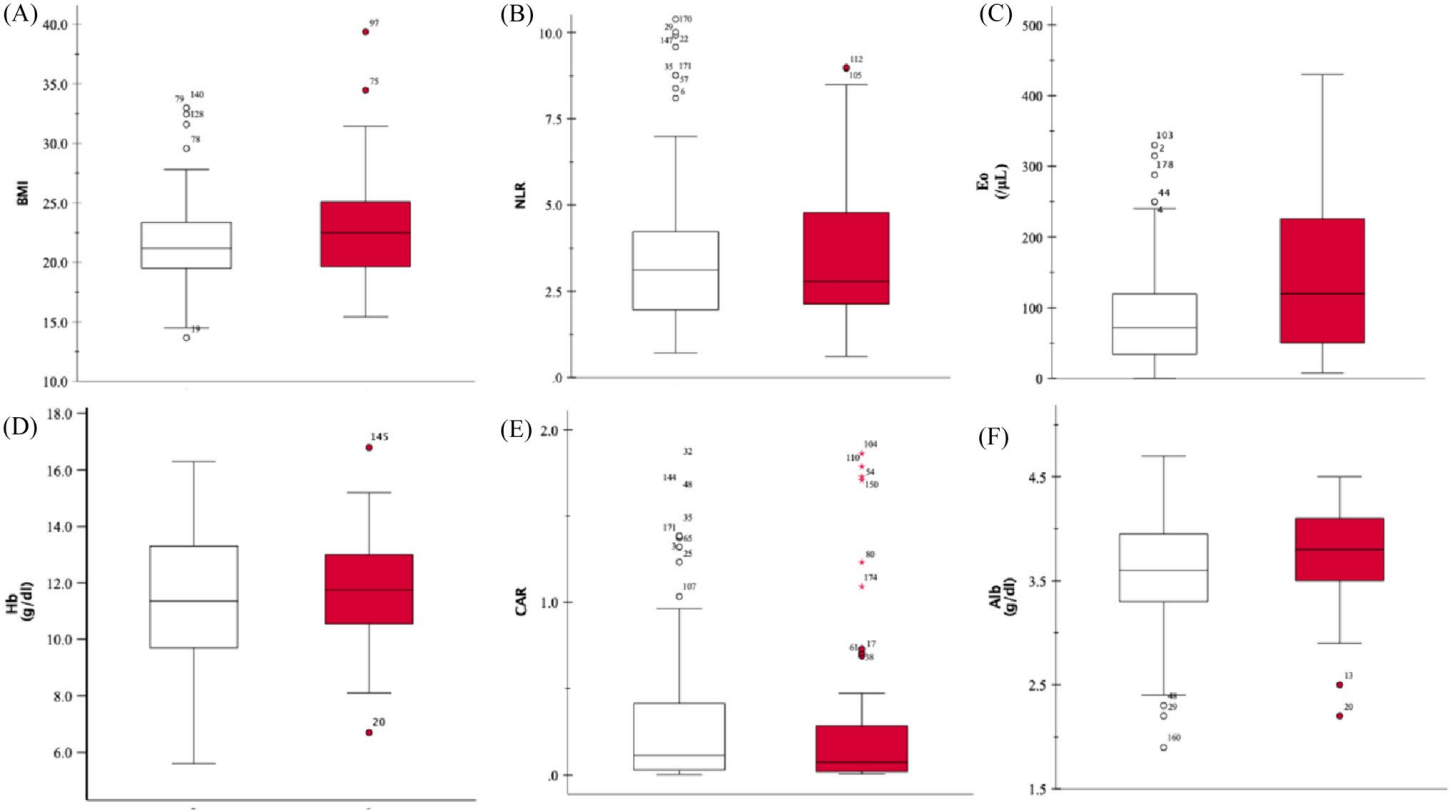
**A–F** Comparison of the nutritional status and laboratory values of the blood between groups with and without irAEs (white box: without irAEs; red box: with irAEs). Boxplot showing the distribution of **A** body mass index (BMI), **B** neutrophil/lymphocyte ratio (NLR), **C** eosinophil count (Eo), **D** hemoglobin (Hb), **E** C-reactive protein/albumin ratio (CAR), and **F** albumin (Alb). The horizontal midline of each box represents the median. The bottom and top of each box are indicated. The 25th and 75th percentiles are represented by the ends of the whiskers, indicating the minimum and maximum values of all data, respectively

**Fig. 2:**
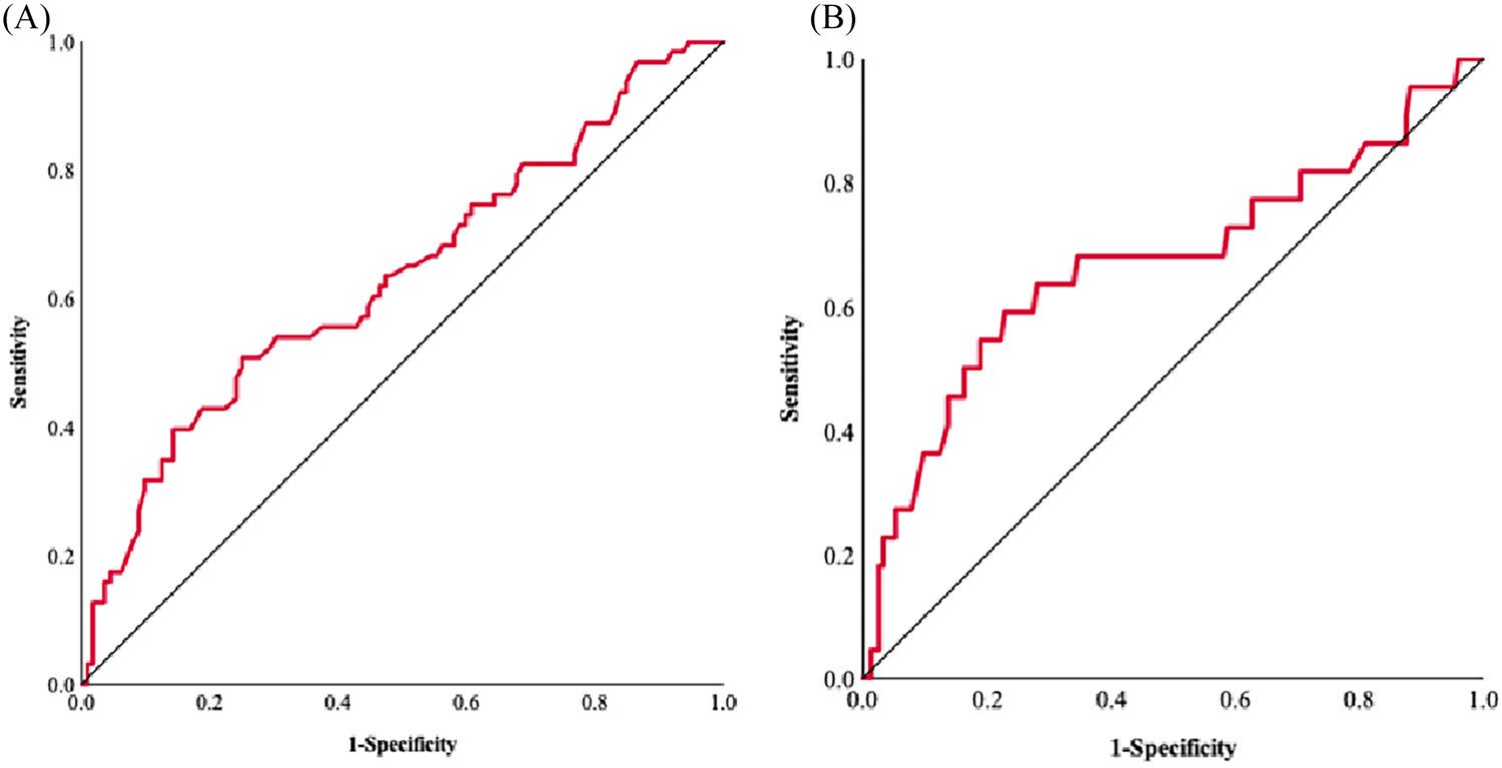
**A**, **B** Receiver operating characteristic curve analysis of the eosinophil counts for the incidence of **A** irAEs and **B** Grade 3 or higher events

Nutritional indices, such as BMI, and hematological laboratory values, such as eosinophil count, were compared between the no-irAE and yes-irAE groups, and the results are depicted in Fig. 1. Significant differences were observed in BMI, eosinophil count, and Alb level, with *p*-values of 0.34, 0.004, and 0.03, respectively. Similarly, ORs for the incidence of irAEs were analyzed using univariate and multivariate analyses. Table 3 shows that eosinophil count was a significant risk factor for the occurrence of irAEs and Grade 3 or higher events (OR 1.01, 95% CI 1.00–1.01, *p*-value 0.02, and OR 1.01, 95% CI 1.00–1.01, *p*-value 0.01, respectively). In addition, we focused on eosinophil count and identified its cutoff value for the incidence of irAEs. Using the ROC curve in Fig. 2, we determined the cutoff value to be 118 (/μL) (area under the curve [AUC] = 0.63, 95% CI 0.55–0.72, *p*-value = 0.004, sensitivity = 0.51, specificity = 0.75). ROC analysis for the incidence of irAEs and Grade 3 or higher events calculated AUC as 0.67 (95% CI 0.53–0.81, *p*-value = 0.01).

**Table 3:** A, B, C. Risk factors for (A) irAEs and (B) Grade 3 or higher events in univariate and multivariate logistic regression analyses, and (C) details of irAEs in two groups divided by eosinophil count values

Variables	Univariate analysis			Multivariate analysis		
	HR	95% CI	*p*-value	HR	95% CI	*p*-value
(A)						
BMI	1.09	1.00–1.20	0.05	1.09	1.00–1.19	**0.04**
NLR	1.08	0.96–1.21	0.20			
Eosinophil	1.00	1.00–1.01	**0.03**	1.01	1.00–1.01	**0.02**
Hb	0.99	0.83–1.18	0.87			
CAR	0.87	0.16–4.59	0.87			
Alb	2.23	0.91–5.48	0.08			
(B)						
BMI	0.92	0.82–1.03	0.15			
NLR	0.82	0.71–0.95	**0.01**	1.20	1.06–1.34	**0.01**
Eosinophil	1.00	0.99–1.00	**0.02**	1.01	1.00–1.01	**0.01**
Hb	0.95	0.73–1.24	0.71			
CAR	1.03	0.16–6.75	0.98			
Alb	0.68	0.18–2.61	0.57			

Variable	*N* = 175 (%)		*p*-value			
	Low-Eo *N* = 115	High-Eo *N* = 60				
(C)						
irAE, any	31 (27)	32 (53)	** < 0.01**			
irAE, Grade 3–4	8 (7)	14 (23)	** < 0.01**			
irAE leading to discontinuation	11 (14)	12 (29)	0.05			
Specific irAE						
Dermatitis	11 (10)	9 (15)	0.32			
Hepatitis	6 (5)	9 (15)	**0.04**			
Pneumonitis	6 (5)	9 (15)	**0.04**			
Colitis	2 (2)	6 (10)	**0.02**			
Endocrine disorder	6 (5)	3 (5)	1.00			
Nephritis	2 (2)	1 (1)	1.00			
Other	5 (4)	6 (10)	0.19			
Multiple irAEs	5 (4)	9 (15)	**0.02**			

HR; Hazard Ratio, CI; confidence interval, BMI; body mass index, NLR; neutrophil/lymphocyte ratio, Hb; hemoglobin, CAR; C-reactive protein/ albumin ratio, Alb; albumin, Eo; eosinophil count

Items with *p*-values in bold were less than 0.05

### Association between eosinophil count and the irAEs

Using the previous cutoff value for eosinophil count, we divided the patients into two groups: low-Eo and high-Eo (*n* = 115 and *n* = 60, respectively). Details of the irAEs are presented in Table 3C. The incidence of irAEs and Grade 3 or higher events was higher in the high-Eo group than in the low-Eo group (53% vs. 27%, *p* < 0.01, and 23% vs. 7%, *p* < 0.01, respectively). The onsets of hepatitis, pneumonitis, and colitis were more frequent in the high-Eo group than in the low-Eo group. Moreover, more patients experienced multiple irAEs in the high-Eo group than in the low-Eo group (15% vs. 4%, *p*-value = 0.02).

## Discussion

We investigated the safety profile of irAEs in patients who were ≥ 80 years and clarified the elevation of eosinophil counts as the risk factor for irAEs. In our study, the incidence of all grades of irAEs was 35%, with a 13% incidence of Grade 3 or higher events. The toxicity-related discontinuation rate due to irAEs was 19%, and the treatment-related hospitalization rate was 5%. Additionally, we found that eosinophil count was a risk factor for the incidence of irAEs. Based on the current cutoffs, the incidence of irAEs in the high-risk population was 53%, and the incidence of Grade 3 or higher events was 27%. Although adverse events should be carefully monitored, IO drugs were safe for the older patients in our study.

A previous study reported a similar incidence of irAEs with IO monotherapy in younger patients, with increased rates of hospitalization and toxicity-related discontinuation [18]. In our retrospective cohort study, irAE details, such as types and incidence rates, were similar to those previously reported in younger participants; the differences were not statistically significant. However, the rate of toxicity-related discontinuation was higher in this study than in a previous study focused on younger patients. The timing of irAE onset was similar to the findings of previous studies [18, 19]. Similar to younger patients, older patients require careful treatment because of the significant variability in the timing of symptom onset. Considering the hospitalization rate, close follow-up is necessary. Medical applications that can detect adverse events remotely are increasingly being developed [20]. However, older patients might encounter challenges in mastering modern applications and media interfaces; therefore, simplifying the system or devising alternative methods becomes necessary [21]. Therefore, a follow-up system that uses human resources such as telephone interviews and visiting nurses may be required.

Based on this study, we propose that Eo is a risk factor for the development of irAEs; however, we examined various other risk factors. When comparing the two groups according to the presence or absence of irAEs, the group without irAEs had significantly more cases of poor PS than the group with irAEs. No decrease in the incidence of toxicity was observed in the poor-PS group. In the PS ≥ 2 group, most patients had shorter survival times, suggesting that an extremely short observation period might have had an effect. Similar to the present study, the relationship between nutritional status and the efficacy and toxicity of IOs has been explored in several studies [17, 22–24]. However, in this study, peripheral blood eosinophil counts showed a stronger correlation with the development of irAEs than with nutritional factors. Previous studies have identified a correlation between peripheral blood eosinophil counts and irAEs, indicating that eosinophil count could be a risk factor for the onset of irAEs independent of patient age [22, 25]. Our research indicates that eosinophil count should be a key laboratory parameter for monitoring when concerns about toxicity arise, particularly in older patients. Although the detailed mechanism of the association between irAEs and eosinophils is unclear, an elevated eosinophil count in older adults may reflect relative adrenocortical dysfunction associated with aging [26–29]. Age-related relative adrenocortical dysfunction may result in decreased cortisol production, altered immune status, and elevated eosinophil count [28, 29]. Since eosinophil counts reflect decreased adrenocortical function, relative corticosteroid deficiency may increase the incidence of irAEs [27]. Cortisol levels were not clearly measured in many cases in this study, and it was impossible to correlate cortisol levels with apparent production levels. In our study, eosinophil counts did not correlate well with any irAE, suggesting that eosinophil counts were associated with a limited number of irAEs, such as pneumonitis. Although the number of cases was limited, an association between multiple irAEs occurring in multiple organs has been suggested. Eosinophil counts can be easily determined using blood tests and should be closely monitored.

Our study has several limitations. This was a retrospective cohort study, and only a few patients were included. The incidence and information about irAEs were collected from medical records and might have been missed if the treating physician's medical records were unavailable.

In conclusion, our study suggests that IO therapy is a safe treatment option for older patients aged > 80 years. Furthermore, caution is required when managing patients with elevated eosinophil counts at the commencement of treatment. Early detection of irAEs and therapeutic intervention is crucial, and future advancements should focus on the development of an appropriate follow-up system, medical devices, and applications that enable the early detection of irAEs in older adults.

## Data Availability

The datasets generated or analyzed during the current study are not publicly available; however, they are available from the corresponding author upon reasonable request.
